# A treatment applying a biomechanical device to the feet of patients with knee osteoarthritis results in reduced pain and improved function: a prospective controlled study

**DOI:** 10.1186/1471-2474-11-179

**Published:** 2010-08-10

**Authors:** Yaron Bar-Ziv, Yiftah Beer, Yuval Ran, Shaike Benedict, Nahum Halperin

**Affiliations:** 1 Department of Orthopedic Surgery, Assaf Harofeh Medical Center, Zerifin, Israel

## Abstract

**Background:**

This study examined the effect of treatment with a novel biomechanical device on the level of pain and function in patients with knee OA.

**Methods:**

Patients with bilateral knee OA were enrolled to active and control groups. Patients were evaluated at baseline, at 4 weeks and at the 8-week endpoint. A novel biomechanical device was individually calibrated to patients from the active group. Patients from the control group received an identical foot-worn platform without the biomechanical elements. Primary outcomes were the WOMAC Index and ALF assessments.

**Results:**

There were no baseline differences between the groups. At 8 weeks, the active group showed a mean improvement of 64.8% on the WOMAC pain scale, a mean improvement of 62.7% on the WOMAC function scale, and a mean improvement of 31.4% on the ALF scale. The control group demonstrated no improvement in the above parameters. Significant differences were found between the active and control groups in all the parameters of assessment.

**Conclusions:**

The biomechanical device and treatment methodology is effective in significantly reducing pain and improving function in knee OA patients.

The study is registered at clinicaltrials.gov, identifier NCT00457132, http://www.clinicaltrials.gov/ct/show/NCT00457132?order=1

## Background

Osteoarthritis (OA) is a major cause of disability in the older population [1], affecting nearly 21 million individuals in the United States alone [2]. Currently there is no cure for OA and treatment is focused on reducing pain and improving function [3].

There is a growing awareness of the importance of biomechanical factors in the pathogenesis and progression of knee osteoarthritis [4-6]. Studies have demonstrated a clinical association between loads, such as lifelong physical work [7], competitive sports [8,9], and obesity [10], and the formation and progression of osteoarthritis [11]. These factors, together with the morphological changes in the musculoskeletal system that occur with age, affect the osteochondral structures [12-15] and neuromuscular control [16]. Neuromuscular control plays a significant part in determining the function and stability of the synovial joint [17] and in mediating the biomechanical structure of articular cartilage [18]. Impairment of the neuromuscular control system contributes to the pathogenesis of osteoarthritis by altering joint biomechanics and causing increased cartilage damage [19,20].

Two main types of non-surgical biomechanical interventions are available for reducing pain and improving function in patients with knee osteoarthritis. The logic behind the first type of intervention is unloading the diseased articular surface by means of wedge insoles, foot orthoses and, more recently, by valgus braces [21-25]. The logic behind the second type of intervention is to improve neuromuscular control for the affected limb, thereby achieving transition from co-activation to coordinated motor response [26]. The key element in this intervention, as in any motor learning, is repetitive exposure of the individual to the desired movement experience [27]. The focus is on training under perturbation in closed kinematic chain movements in which the whole limb, rather than just a single joint, is regarded as a kinetic functional unit. This approach, however, showed encouraging results only in case studies [28]. Recently, a novel foot-worn biomechanical device that incorporates the logic of both types of non-invasive interventions was examined by Haim et al. using a three-dimensional gait analysis [29]. This device has the capability to change the location of the center of pressure (COP) during walking, hence it can shift the external forces acting on the body. Furthermore, the device generates perturbation during movement that challenges neuromuscular control.

The purpose of the current study is to examine the effectiveness of an individually calibrated biomechanical device and treatment methodology that combines the logic of both interventions for reducing pain and improving function in knee osteoarthritis patients.

## Methods

### Study Design and Participants

All patients gave written informed consent before entering the study. The protocol was approved by the Institutional Helsinki Committee Registry of Assaf Harofeh Medical Center (Helsinki registration number 44/05 and NIH clinical trial registration number NCT00457132). The study was conducted at the Department of Orthopedics. Eligibility was defined as symptomatic bilateral knee OA of the medial compartment for at least 6 months. All patients fulfilled the American College of Rheumatology clinical criteria for OA of the knee [30] and had radiographically assessed osteoarthritis of the knee according to the Kellgren & Lawrence (K&L) scale [31]. All patients had a varus knee alignment. Exclusion criteria were acute septic arthritis, inflammatory arthritis, patients with a history of increased tendency to fall, patients with a history of knee buckling, lack of physical or mental ability to perform or comply with the treatment procedure, diabetes mellitus, and patients with a history of pathological osteoporotic fracture.

**Figure 1:**
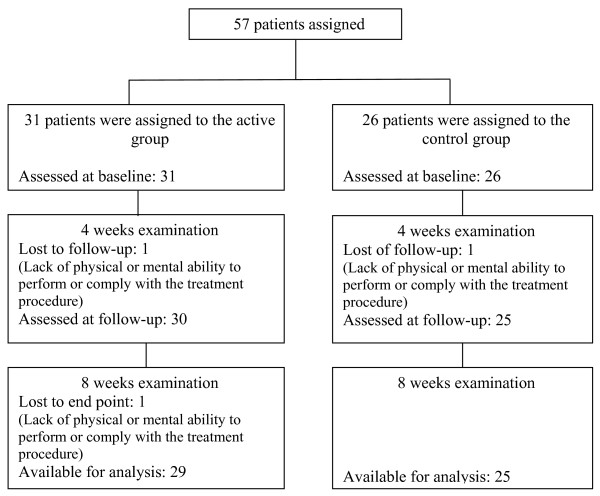
**Patient flow-chart**.

The study was conducted between December 2005 and February 2006. In December 2005, a total of 57 patients were allocated to the study by the senior orthopedic surgeon (N.H.) according to the inclusion and exclusion criteria (Figure 1). Patients that were found eligible were referred to the administrative secretary of the clinic. Patients were then asked to choose one of the two days of the week that were scheduled for the study, according to their own convenience. One of the days was designated for the active group treatment and the other day was designated for the control group treatment. The patients from both groups, the senior orthopedic surgeon, the investigators and the administrative secretary did not know to which group the patients were assigned. Only the therapist applying and calibrating the device knew to which group a patient belonged. Throughout the course of the study patients from one group had no knowledge of the patients from other group. At the first session patients underwent a medical examination conducted by two orthopedic surgeons (Y.R. and S.B) who did not know to which of the two groups the patients were assigned. Following the physical examination patients were referred to the treatment room that was located at a separate facility. The physical therapist who applied the intervention was not aware of the patients' medical condition and clinical examinations results throughout the study.

## Interventions

The biomechanical system (Apos system, AposTherapy - Sports and Medical Technologies Ltd. Herzliya, Israel) is a device combined with a treatment methodology. The device is calibrated to the individual patient according to pathology and motion characteristics. Each patient is asked to walk away from and then back towards the therapist. A visual gait evaluation is carried out by the therapist and the device is appropriately calibrated. Appropriate calibration is defined as bringing the damaged joint to a biomechanical alignment that minimizes/eliminates pain by shifting and/or changing the applied forces and, consequently, altering the pressure distribution within the joint [26,29]. Together with the biomechanical perturbations applied through all phases of the step-cycle (i.e., initial contact, mid-stance and toe-off), this device enables home-based, dynamic, functional and repetitive training intended to improve neuromuscular control.

**Figure 2:**
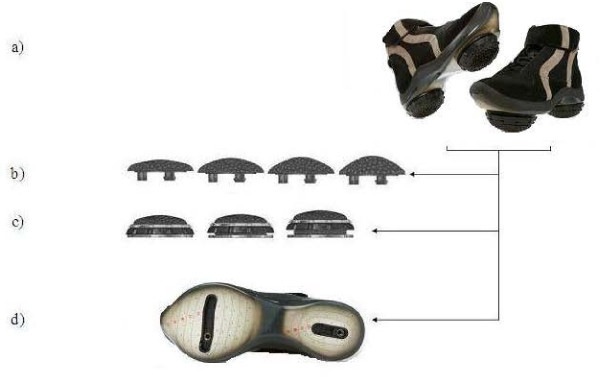
**The biomechanical device**. (a) Bio-mechanical device comprising of two individually calibrated elements and a foot-worn platform which they are attached to under the hindfoot and forefoot regions. (b) Bio-mechanical elements are available in different degrees of convexity and resilience; (c) Height versatile base with 0.25 mm increments; (d) Specially designed sole includes two mounting rails and positioning matrix to enable flexible positioning of each bio-mechanical element.

The device consists of two convex shaped biomechanical elements attached to each of the patient's feet (Figure 2). One is located under the hindfoot region and the other is located under the forefoot region. The elements are attached to the patient's foot using a platform in the form of a shoe. The platform is equipped with a specially designed sole that consists of two mounting rails that enable flexible positioning of each element under each region.

The methodology consists of two phases, the first brings the joint to diminished pain alignment and the second applies perturbations while walking. In medial compartment deformity the element under the hindfoot is shifted laterally from the baseline position. This shifts the COP in the foot laterally, thereby reducing the magnitude of the adduction moment acting on the knee joint [29]. This is done until the patient reports minimal pain during initial contact. The element under the forefoot is shifted medially from the baseline position until the patient reports minimal pain during mid-stance. Once the desired alignment is achieved, the patient should report immediate pain relief while walking. Perturbation is achieved by walking on two convex shaped elements that create controlled instability in gait.

### Treatment Protocol

#### Active group

The device was calibrated to each patient after baseline assessment. Treatment was then initiated and continued daily for a period of eight weeks, during which the device was again recalibrated, as necessary, after the first, third and sixth week. Patients were instructed to follow a treatment protocol based on walking during activities of daily living, starting with 10 minutes of indoor walking each day during the first week and gradually increasing to 30 minutes of daily outdoor walking by the last week. The patients were told to continue their daily activities wearing their regular footwear. Patients were also instructed not to ingest any pain relief medication, non-steroidal anti-inflammatory drug or food supplements starting from 10 days before the study (drug wash-out) except for unmarked acetaminophen tablets that had been given to them at the start of the study as a rescue medication.

#### Control Group

Patients in the control group were fitted with an identical foot worn platform that did not include the biomechanical elements or the movement rails. Without these, the shoe is left with a regular rubber sole. We assumed that once the elements were removed, the shoe acts like any other walking shoe. They were instructed to follow the same treatment protocol as the active group and to not ingest any medication or food supplements apart from the unmarked rescue medication that had been given to them. Therefore, for all intents and purposes, the foot worn platform alone was not intended as a type of intervention. It was a complete control aside from the walking routine of the patients. Both the active group and the control group had exactly the same number of visits. During the visits in the first, third and sixth weeks, patients from both groups walked back and forth along a 12 m line while the physiotherapist technician monitored them walking and noted observations.

### Outcome Measures

We tested the clinical efficacy of a device designed to reduce pain and improve function in knee osteoarthritis patients using the Western Ontario and McMaster Osteoarthritis Index (WOMAC) [32] and the Aggregated Locomotor Function (ALF) [33]. Patients were told to score the questionnaires according to the pain and function of their worse knee according to them. The ALF scale is a sum of mean timed scores (seconds) of three locomotor functions: time taken to walk 8 meters, time taken to ascend and descend 7 stairs and time taken to transfer from sitting to standing. The patients were evaluated three times during the study: at baseline, at 4 weeks and at the 8-week endpoint. Measurements of the ALF test were made with the patients barefoot as required by the outcome protocol.

Secondary outcome measures were the SF-36 [34] health survey and the Knee Society Score [35], the latter of which includes a physical examination subscale.

### Statistical Analysis

The criteria for clinical response to a treatment had been defined by the Outcome Measures in Rheumatology Clinical Trials (OMERACT) and Osteoarthritis Research Society International (OARSI). They are either an improvement in pain or in function of at least 50 percent with a decrease of 2.0 cm on the visual-analogue-scale (VAS) for pain or function, or an improvement in both pain and function of at least 20 percent with a decrease of 1.0 cm on the VAS [36]. The trial was designed to have 80% power with a two-sided p = 0.05 level to detect a mean difference of 2.0 cm (corresponding to means of 2.0 cm versus 4.0 cm and the common within-group standard deviation of 2.5 cm) on the WOMAC VAS scale (0-10 cm), and a difference of -10.0 sec (corresponding to means of 30.0 sec versus 40.0 sec and the common within-group standard deviation of 10.0 sec) on the ALF scale. Assuming a small number of dropouts during follow-up, we recruited 57 patients to accommodate the needed sample size for each of the two groups. The sample size was defined according to a power calculation that tested (2-tailed) the null hypothesis that the two population means were equal (i.e., that the study will have power of 86.1% and 96.8% for WOMAC and ALF, respectively, to yield a statistically significant result).

To avoid various misleading artifacts we checked our hypotheses based on the intention to treat (ITT) analysis. In order to reject baseline differences between the groups we examine the hypothesis that the two groups were comparable at baseline by using an independent (2-tailed) t-test on patient age, sex, Kellgren & Lawrence grade and the baseline scores of all four outcomes. Changes within the groups and differences between the groups in primary and secondary outcomes were calculated by repeated measures ANOVA, which produced three tests of significance: difference in changes over time between groups, total changes over time and difference between groups in general. These three tests are appropriate for examining our hypothesis that assumes: a) clinically significant improvement in scale for the active group; b) no clinically significantly improvement in scale for the control group; c) no advantage in scale at baseline for the active group compared to the control group. The analysis was performed by an external statistician using SPSS software (SPSS, Chicago).

## Results

### Participants

Of the 57 patients participating in the study, 31 (8 males, 23 females, aged 64 ± 8.1 years) were assigned to the active group and 26 (7 males, 19 females, aged 66.03 ± 7.8 years) to the control group. Three patients were lost to follow-up, leaving 29 patients in the active group and 25 patients in the control group available for analysis (Figure 1). The patient characteristics and baseline results for measured variables were similar between both groups. The study population characteristics are presented in Table 1.

**Table 1:** Patients Characteristics of the Two Study Groups*

Characteristic	Active (N = 31)	Control (N = 26)	P Value†
Age	64.0 ± 8.1	66.0 ± 7.8	0.31
Female - no. (%)	23 (74)	19 (73)	
Kellgren and Lawrence (K&L)			
K&L 2 - no. (%)	3 (10)	7 (27)	0.155
K&L 3 - no. (%)	11 (36)	5 (19)	
K&L 4 - no. (%)	17 (55)	14 (54)	
Body Mass Index (BMI)	30.3 ± 4.30	29.7 ± 3.79	0.56

* Mean values are presented as mean ± SD

† P values for the difference between the groups' baseline characteristics.

There were no significant differences between the groups at baseline

### Clinical Outcomes

At the 8-week endpoint the WOMAC pain score and function score revealed significant differences between the groups over time (Time by treatment interaction, p < 0.001). The active group reported significant pain relief after 8 weeks of treatment with a mean difference of 3.5 cm (64.8%) and a 95% confidence interval ranging between 2.7-4.4. In contrast, the control group reported no pain relief, having a mean increase of 0.4 cm (8%) with a 95% confidence interval ranging between -1.7-0.8. On the WOMAC function scale, the active group reported significant improvement with a mean decrease of 3.2 cm (62.7%) after 8 weeks and a 95% confidence interval ranging between 2.5-4.1. The control group reported no function improvement, having a mean increase of 0.5 cm (9.8%) with a 95% confidence interval ranging between -1.4-0.5. The extent of improvement in the level of pain and function corresponds with the OMERACT criteria for clinical response to treatment.

Furthermore, the ALF final mean score values demonstrated significant differences between the groups over time (p < 0.001). The active group showed significant improvement in function with a mean decrease of 11.6 sec. (31.4%) and 95% confidence interval ranging between 8.7-14.5 on the ALF scale after 8 weeks. No improvement was shown by the control group, having a mean decrease of 0.7 sec (1.8%) and 95% confidence interval ranging between -0.9-2.1 after 8 weeks. Table 2 summarises the results.

**Table 2:** Mean values and time by treatment interaction results of primary outcome measures*

Outcome	Baseline	Follow-up (4 weeks)	Final (8 weeks)	P Value†
WOMAC - Pain				
Active	5.4 ± 2.7	3.1 ± 2.2	1.9 ± 1.6	<0.001
Control	5.0 ± 2.7	5.1 ± 2.2	5.4 ± 2.7	
P Value	0.50	0.002	<0.001	
WOMAC - Stiffness				
Active	5.7 ± 3.0	3.7 ± 2.5	1.9 ± 2.3	<0.001
Control	5.4 ± 3.3	5.4 ± 3.0	5.2 ± 3.2	
P Value	0.67	0.02	<0.001	
WOMAC - Function				
Active	5.1 ± 2.6	3.1 ± 1.9	1.9 ± 1.5	<0.001
Control	5.2 ± 2.3	5.5 ± 2.2	5.7 ± 2.6	
P Value	0.91	<0.001	<0.001	
WOMAC - Total Score				
Active	5.4 ± 2.6	3.3 ± 2.0	1.9 ± 1.7	<0.001
Control	5.2 ± 2.6	5.3 ± 2.3	5.4 ± 2.6	
P value	0.71	0.001	<0.001	
ALF Score				
Active	36.9 ± 11.5	29.2 ± 8.8	25.3 ± 6.6	<0.001
Control	39.2 ± 16.7	37.7 ± 15.5	38.5 ± 16.3	
P value	0.56	0.01	<0.001	

* Primary outcome measures: Western Ontario and McMaster Osteoarthritis Index (WOMAC) questionnaire (0-10 cm Scale) and Aggregated locomotor function (ALF) (sec.). Mean values are presented as mean ± SD.

**† **The vertical p-value represent the time by treatment interaction results. This represents the difference between the groups at different points in time. The horizontal p-value represents group differences in the three examination points. This represents the differences in the active group across the three follow-ups. No significant differences were found in the control group.

The two secondary outcomes, SF-36 health survey and the Knee Society Score, showed significant time by treatment interaction. That is to say, significant changes were found within the active group after 4 weeks and after 8 weeks and significant differences were found between the groups at 4 weeks and at 8 weeks. Table 3 summarises the results. In addition, no side effects were reported by any of the patients.

**Table 3:** Mean values and time by treatment interaction results of secondary outcome measures*

Outcome	Baseline	Follow-up (4 weeks)	Final (8 weeks)	P Value†
SF-36 Physical Functioning subscale				
Active	46.0 ± 18.6	61.8 ± 19.2	69.2 ± 21.0	P < 0.001
Control	43.7 ± 21.1	36.7 ± 20.9	38.7 ± 22.1	
P Value	0.66	<0.001	<0.001	
SF-36 Mental Health subscale				
Active	57.5 ± 45.3	73.6 ± 38.2	90.8 ± 23.4	0.004
Control	56.0 ± 39.3	42.7 ± 40.3	44.0 ± 39.3	
P value	0.90	0.006	<0.001	
SF-36 Score Results				
Active	56.0 ± 21.1	68.1 ± 17.7	77.1 ± 15.1	<0.001
Control	53.5 ± 18.9	51.1 ± 19.5	48.5 ± 22.1	
P value	0.65	0.001	<0.001	
Knee Society Score				
Knee Score				
Active	54.1 ± 17.9	67.0 ± 14.3	78.6 ± 11.6	<0.001
Control	60.3 ± 17.9	62.1 ± 17.8	50.8 ± 17.8	
P value	0.44	0.25	<0.001	
Knee Function Score				
Active	52.6 ± 17.6	61.8 ± 16.8	71 ± 16.8	<0.001
Control	56.0 ± 14.8	56.4 ± 17.0	51.4 ± 16.9	
P value	0.20	0.23	<0.001	

* Secondary outcome measures: SF-36 Health Survey (0-100 scale) and knee society score (0-100 scale). Mean values are presented as mean ± SD.

**† **The vertical p-value represent the time by treatment interaction results. This represents the difference between the groups at different points in time. The horizontal p-value represents group differences in the three examination points. This represents the differences in the active group across the three follow-ups. No significant differences were found in the control group.

**Figure 3:**
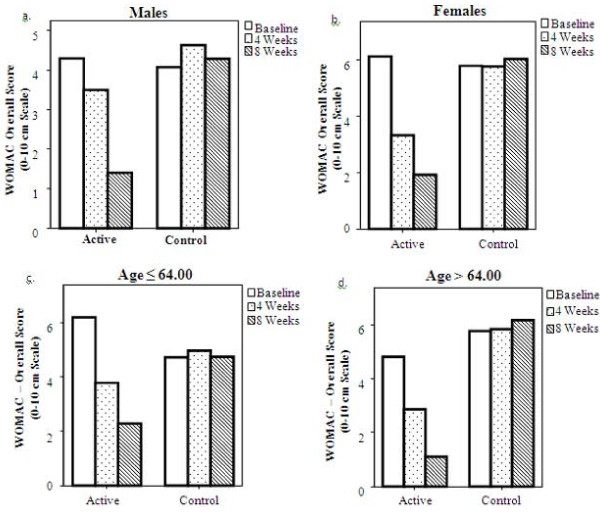
**Western Ontario and McMaster Osteoarthritis Index (WOMAC) over score mean values - distribution by age and gender**. Time by treatment effect was significant in the active group in all 4 conditions. There was no significant time by treatment effect in the control group. P < 0.05

We further investigated the changes in the primary outcomes and secondary outcomes for both groups in distribution of age and gender. Time by treatment interaction was significant for the active group in the three measured parameters for gender and age: 1) WOMAC overall score - males p = 0.009, females p < 0.001, under the age of 64 p < 0.001, above the age of 64 p < 0.001; 2) ALF overall score - males p < 0.001, females p < 0.001, under the age of 64 p < 0.001, above the age of 64 p < 0.001; 3) SF-36 overall score - males p = 0.04, females p = 0.001, under the age of 64 p < 0.001, above the age of 64 p < 0.001. There was no time by treatment effect in the control group. Figure 3 illustrate the effect of the treatment between genders and between ages in the WOMAC results.

Patients from the control group use more of the rescue medication given to them at the start of the study than did the active group. After 4 weeks, the active group as a whole consumed 145 rescue pills whereas the control group consumed 281 pills. After 8 weeks, the active group consumed 128 pills and the control group consumed 366 pills. Overall the active group consumed 273 pills and the control group consumed 647 pills.

## Discussion

The results of the current study demonstrate the clinical efficacy of the individually fitted biomechanical device and treatment methodology in reducing pain and improving function in knee osteoarthritis patients. The device we describe combines "unloading" [21-23] the damaged compartment and "perturbation" [27,28] (stimulation) of neuromuscular control, two types of interventions that are recommended for patients with knee OA. The interpretation of our results can therefore be based on earlier works.

Past studies showed and stressed the importance and efficacy of unloading the diseased articular surface in patients with knee OA [21-23]. A recent study showed that the biomechanical device used in this study can not only accomplish this but can also reduce the external adduction moment loads acting on the knee joint in healthy population [29]. Furthermore, Fitzgerald et al. demonstrated the importance of "perturbation" intervention [26,28]. The purpose of the "perturbation" intervention is to stimulate neuromuscular control of the affected limb. Lewek et al. explained that the aim is to shift from global concentric contraction muscle patterns to a coordinative motor response [4]. This should be done by repetitive exposure to perturbation in closed kinematic chain movements.

The key feature of the new device in this study is repetitive perturbations with diminished pain in the patient's own environment and during ADL. The structure of the biomechanical elements and the treatment methodology promotes perturbations throughout all phases of step cycle. The perturbations are repeated thousands of times during walking and activities of daily living, influencing the whole kinetic and kinematic chain. In our study we equipped the device with low convexity biomechanical elements in order to promote only mild perturbations and enable the patients to walk in a controlled manner.

Usually, patients who exercise while they are in pain adopt pathological patterns and also fail to comply [37]. In the current study, the patients reported diminished pain or no pain while using the device immediately after calibration. This relief in pain enabled them to walk painlessly with the device and presumably reacquire proper neuromuscular control skills [21-23] and appropriate motor patterns that they can maintain when not using the device. In addition, unlike stationary devices, the new device enables rehabilitation during the patient's daily life activities and in the patient's environment, where compliance can be expected to be higher.

The study was designed to be a prospective, double blind, sham controlled study. Maintaining a sham group of patients with advanced knee OA for a period longer than two months is problematic for several reasons. First, from an ethical aspect, the control group patients were in pain and without treatment. Second, control group compliance was expected to lessen after a period of two months due to pain. As shown and explained above, the intervention group experienced a significant reduction in pain and improvement in function. In contrast, the control group showed no reduction in pain and a slight deterioration in function. The lack of improvement in pain and the deterioration in function in the control group can be explained when considering the patient population, exercise extent and their clinical situation. Our patients had moderate-to-severe knee osteoarthritis, were not allowed to use NSAIDS and food supplements, and were asked to follow a treatment program based on dynamic joint loading without biomechanical intervention.

This study lacked randomization in the assignment of the patients to control and active groups. Although both the active and the control group were similar in their characteristics and in the measured variables at baseline, future studies should implement a randomization procedure in assigning patients to control and active groups.

Patients in our study were told not to consume any medications aside from the rescue pills given to them at the start of the study. This was done in order to evaluate the effectiveness of the device as a stand-alone treatment without the use of any other interventions or medications. Since we cannot ask patients to refrain from taking medications for a long period of time, we made our study only 8 weeks.

The control group in our study did not demonstrate any placebo effect. This may be explained in two ways. First, because according to the protocol the control group was told to walk with the device even while in pain, we assumed that this worsened their symptoms and balanced out any placebo effect. Second, it may be that any placebo effect only occurred in the first two weeks and as a result our first evaluation at four weeks did not capture the placebo effect.

The results of this study introduce a device that resulted in significant reduction of pain and function, which is the focus of knee OA treatment. As such, this device and methodology may be a possible treatment for patients with knee OA. Future studies should examine the long term effect of the device on patients with medial compartment knee OA and patients with other musculoskeletal pathologies.

## Conclusion

Our data show that using the AposTherapy system and treatment methodology is an effective non-pharmacological therapy that improves both pain and function in patients with knee OA.

## Competing interests

The authors declare that they have no competing interests.

## Authors' contributions

All authors read and approved the final version of the manuscript.

YBZ and YB are responsible for conception and design, revising the article and final approval of the manuscript. YR and SB are responsible for data collection, drafting the article and final approval of the manuscript. NH is responsible for revising the article and final approval of the manuscript.

## Pre-publication history

The pre-publication history for this paper can be accessed here:

http://www.biomedcentral.com/1471-2474/11/179/prepub
